# Imaging of COVID-19 simulators

**DOI:** 10.1186/s43055-020-00379-9

**Published:** 2021-01-05

**Authors:** Abdelghany Mohammed Motawea, Suzan Omar, Rabab Yasin

**Affiliations:** Radiology Department, Menofia University Faculty of Medicine, Menofia, Shibin Al Kawm, Egypt

**Keywords:** COVID-19 coronavirus infections, CT computed tomography, Atypical pneumonia, Interstitial lung diseases

## Abstract

**Background:**

Coronavirus (COVID-19) pneumonia emerged in Wuhan, China, in December 2019. It was highly contagious spreading all over the world, with a rapid increase in the number of deaths. The reported cases have reached more than 14 million with more than 600,000 deaths around the world. So, the pandemic of COVID-19 became a surpassing healthcare crisis with an intensive load on the healthcare resources.

In this study, the aim was to differentiate COVID-19 pneumonia from its mimickers as atypical infection, interstitial lung diseases, and eosinophilic lung diseases based on CT, clinical, and laboratory findings.

**Results:**

This retrospective study included 260 patients, of which 220 were confirmed as COVID-19 positive by two repeated RT-PCR test and 40 were classified as non-COVID by two repeated negative RT-PCR test or identification of other pathogens, other relevant histories, or clinical findings.

In this study, 158 patients were male (60.7 %) and 102 patients were female (39.3%). There was 60.9% of the COVID-19 group were male and 39.1% were female. Patients in the non-COVID group were significantly older (the mean age was 46.4) than those in the confirmed COVID-19 group (35.2y). In the COVID-19 group, there was exposure history to positive cases in 84.1% while positive exposure history was 20% in the non-COVID group.

**Conclusion:**

The spectrum of CT imaging findings in COVID-19 pneumonia is wide that could be contributed by many other diseases making the interpretation of chest CTs nowadays challenging to differentiate between different diseases having the same signs and act as deceiving simulators in the era of COVID-19.

## Background

In December 2019, the first case with acute lower respiratory tract infection caused by the novel coronavirus (nCoV-2019) was reported in China [1]. In March 11, 2020, the World Health Organization has announced that coronavirus disease 2019 (COVID-2019) is a pandemic and public health emergency of a global scope [2]. In April 8, 2020, the epidemic had reached to the whole earth countries, and the first million of infected humans had been reached [3].

Clinically, COVID-19 has variable presentations from an asymptomatic infection or mild upper respiratory tract symptoms to vigorous viral pneumonia with respiratory failure and even death [4].

At the time of writing these words, the reported cases have reached more than 14 million with more than 600,000 deaths around the world. So, the pandemic of COVID-19 became a surpassing healthcare crisis with an intensive load on the healthcare resources [5].

This rapid wide explosion of the pandemic was due to the lack of the early detection and control of the infection. Also, not all patients are tested, especially asymptomatic cases, or cases with mild symptoms. The primary standard of COVID-19 confirmation is the microbiological tests namely real-time polymerase chain reaction (RT-PCR) [6].

Computed tomography (CT) is used as an important complementary tool to RT-PCR for diagnosing COVID-19 in this ongoing pandemic because these microbiological tests may be unavailable or slow in this emergency context [7].

As RT-PCR may be insufficient, shortage of kits, falsely negative, extended processing period, or long mean time interval between initial negative and positive lab tests as well as its variable sensitivity ranging from 37 to 71%, the chest CT shows a sensitivity of 97% in the diagnosis of COVID-19 [8].

So, the chest CT can play a main role in the early detection and management of COVID-19 pneumonia [6], especially for patients who have symptoms for more than 3 days [9].

Now, the systems of typical chest CT findings in COVID-19 have been established mainly involving the bilateral, peripheral, and basal predominant ground-glass opacities (GGOs) with or without consolidation with a peak of 9–13 days after the infection [10].

Also, atypical chest CT findings like central apical predominance, cavitations, masses, nodules, tree-in-buds, lymphadenopathy, and pleural thickening are included in the spectrum of imaging findings [11].

The spectrum of CT imaging findings in COVID-19 is wide and includes a lot of signs that could be contributed by many other diseases making the interpretation of chest CTs nowadays like an imitation game, for instance, not every GGO is a COVID-19, so it is a challenging to differentiate between different diseases having the same signs and act as deceiving simulators in the era of COVID-19.

In this study, the aim was to differentiate COVID-19 pneumonia from its mimickers based on CT findings, clinical, and laboratory details.

## Methods

Two-hundred sixty patients were enrolled in this retrospective study in the period of the 30th of June and the 15th of August 2020. There were 158 males (60.7%) and 102 females (39.3%) with male to female distribution was 1.5:1.

All patients underwent high-resolution CT within 7 days after the onset of respiratory symptoms. Of those 260 patients, 220 cases of COVID-19 were confirmed with two consecutive positive PCR tests.

### Inclusion criteria include

Patients presented with fever and/or respiratory symptoms within 7 days of CT examination and/or exposure history to confirmed cases or respiratory symptom-related patient.

The clinical data including the age, sex, exposure history, nasopharyngeal swab results, and laboratory parameters of all patients were collected.

The study protocol was approved by the local ethics committee. All patients provided a written informed consent.

### CT image acquisition

CT scans were performed within 7 days after symptom onset on a helical 64-slice CT Philips (parameters Kv 120, mAs 200, field of view 350 mm, thickness 0.67 mm, increment 0.67 mm matrix 768, scan time 5.6 s). Image reconstruction was done at a slice thickness of 1–1.25 mm.

### CT image analysis

The image analysis of each patient described:

- A number of lobes involved.
- Distribution characteristics of the lesions (e.g., peripheral distribution, central distribution, subpleural distribution, and posterior distribution).
- A pattern of the lesion (e.g., ground glass opacification (GGO) with or without consolidation, crazy-paving pattern, and the shape of the GGO).
- Associated signs in the lesion (e.g., bronchial and/or bronchiolar wall thickening), and tree-in-bud sign).

Based on the CT findings, the level of suspicion of COVID-19 infection is graded from very low or CO-RADS 1 up to very high or CO-RADS 5.

CORADS 1: The CT is normal or there are findings that indicate a non-infectious disease like congestive heart failure, sarcoid, histoplasmosis, malignancy, UIP, or fibrotic NSIP.

CORADS 2: Level of suspicion of COVID-19 infection is low. CT findings consistent with other infections like typical bronchiolitis with tree-in-bud and thickened bronchus walls.

CORADS 3: COVID-19 unsure or indeterminate. CT abnormalities indicating infection, but unsure whether COVID-19 is involved, like widespread bronchopneumonia, lobar pneumonia, and septic emboli with ground glass opacities

CORADS 4: The level of suspicion is high. Mostly, these are suspicious CT findings but not extremely typical:

- Unilateral ground glass
- Multifocal consolidations without any other typical finding
- Findings suspicious of COVID-19 in underlying pulmonary disease.

CORADS 5: The level of suspicion is very high. CT findings: bilateral GGO and consolidation, basal preference, vascular thickening, and subpleural bands. PCR is positive.

Patients with low CORADS score (1 to 3) and patients with CORADS 4 with negative PCR test were the main concern. All clinical and laboratory data were meticulously analyzed to reach the final diagnosis.

Three experienced radiologists (20 and 15 years of experience) independently reviewed all the scans. There was a perfect inter-observer agreement between the readers as regards CORADS scoring of the cases (*K* = 0.83).

### Statistical analysis

Inter-observer agreement analysis was performed by the Cohen coefficient was to determine segmental lesion detection consistency among observers. Values were interpreted on the basis of the convention by Landis and Koch as follows: Kappa agreement ˂ 0 less than chance agreement, 0.01–0.20 slight agreement, 0.21–0.40 fair agreement, 0.41–0.60 moderate agreement, 0.61–0.80 substantial agreement, and 0.81–0.99 almost perfect agreement.

## Results

This retrospective study included 260 patients, of which 220 were confirmed as COVID-19 positive by two repeated RT-PCR test, and 40 were classified as non-COVID by two repeated negative RT-PCR tests or identification of other pathogens, other relevant histories, or clinical findings.

In this study, 158 patients were male (60.7%) and 102 patients were female (39.3%). There were 60.9% of the COVID-19 group who were male and 39.1% who were female. Patients in the non-COVID group were significantly older (the mean age was 46.4) than those in the confirmed COVID-19 group (35.2 years). In the COVID-19 group, there was exposure history to positive cases in 84.1% while positive exposure history was 20% in the non-COVID group.

The duration between the start of symptoms and CT imaging was about 3 days in the COVID-19 group and about 5 days in the non-COVID group (Table 1).

**Table 1:** Demographic data of the cases

Parameter	COVID confirmed cases	Low CORAD score and COVID non-confirmed cases
**Age**	35.2 (22–61)	46.4 (10–63)
**Gender**		
**Male**	134=60.9%	24=60%
**Female**	86=39.1%	16=40%
**Exposure history**	185=84.1%	8=20%
**Duration between symptoms and CT imaging**	3 (1–6) days	5 (1–7)

The most presenting symptoms (Table 2) in the COVID-19 group were fever (95.4%) and cough (92.7%) while the most presenting symptom in the non-COVID group was cough in 92.5%.

**Table 2:** Clinical presentation of the cases

Presenting symptoms	COVID confirmed cases		Low CORAD score and COVID non-confirmed cases	
	***n***=220	%	***n***=40	%
**Fever**	210	95.4	25	62.5
**Cough**	204	92.7	37	92.5
**Sputum**	60	27.3	33	82.5
**Runny nose**	170	77.3	16	40
**Loss of smell and taste**	190	86.4	5	12.5
**Chest pain/tightness**	168	76.4	28	70
**Sore throat**	148	67.3	3	7.5
**Diarrhea and abdominal pain**	126	57.3	2	5
**Fatigue and muscle pain**	180	81.8	14	35
**hemoptysis**	0	0	1	0.025

In the non-COVID group, 55% had a high leucocytic count; however, in the COVID-19 group, 76.4% had a low leucocytic count. COVID-19 patients had a high ferritin level (86.4%) and high a D-dimer level (97.1%); however, the non-COVID group had predominant normal ferritin level (72.5%) and normal D-dimer (95%) (Table 3).

**Table 3:** Laboratory finding in the cases

Laboratory finding	COVID confirmed cases		Low CORAD score and COVID non-confirmed cases	
	***n***=220	%	***n***=40	%
**Leucocytic count**				
**High**	16	7.3	22	55
**Low**	168	76.3	11	27.5
**Normal**	36	16.4	7	17.5
**Ferritin**				
**High**	190	86.4	4	10
**Low**	10	4.5	7	17.5
**Normal**	20	9.1	29	72.5
**d-dimer**				
**High**	174	79.1	2	5
**Low**	14	6.4	-	0
**Normal**	32	14.5	38	95

### CT imaging findings (Table 4)

A high-resolution CT chest was done for all 260 examined patients. GGO with or without consolidation was the constant imaging feature seen in both groups. Rounded GGO was more prominent in the COVID-19 group (*n* = 194, 88.2%). Bilateral involvement with peripheral and lower lobe predominance was more evident in the COVID-19 group. Bronchial wall thickening and central predominance were more prominent in the non-COVID group (42.5% and 27.5%, respectively). Tree-in-bud opacity and cavitations were seen only in the non-COVID group. Table 5 representing the diagnostic clues for COVID-19 mimicker based on CT, laboratory, and clinical findings.

**Table 4:** CT findings in all cases

CT imaging findings	COVID confirmed cases		Low CORAD score and COVID non-confirmed cases		Total
	***n***=220	%	***n***=40	%	
**Bilateral involvement**	210	95.5	31	77.5	241
**Peripheral distribution**	188	85.5	22	55	210
**Lower lobe predominance**	210	95.5	28	70	238
**Ground glass +/− consolidation**	220	100	40	100	260
**Rounded ground glass opacities**	194	88.2	3	7.5	197
**Crazy paving**	172	78.2	4	10	176
**Subpleural bands**	148	67.3	12	30	160
**One lobe involvement**	6	2.7	5	12.5	11
**Central predominance**	14	6.4	11	27.5	25
**Tree in bud**	0	0	12	30	12
**Bronchial wall thickening**	44	20	17	42.5	61
**cavitation**	0	0	5	12.5	5
**Pleural effusion**	4	1.8	12	30	16

**Table 5:** Diagnostic clues for COVID-19 mimicker

Final diagnosis		Diagnostic clues				Number of cases
		Clinical history	Pleural/cardiac involvement	Pulmonary parenchymal involvement	Laboratory/biopsy	
**Bronchial asthma**		History of bronchial asthma		Emphysematous changes Subpleural sparing Bronchiactasis Peribronchial thickening Centrilobular nodules due to superadded infection		3
**Eosinophilic lung**	Churg-Strauss syndrome	History of bronchial asthma, sinusitis	Pleural effusion		Peripheral eosinophilia	1
	Drug rash with eosinophilia and systemic symptoms (DRESS) syndrome	Clinical history, skin rash	Pleural effusion		Peripheral eosinophilia	1
	Loffler syndrome			Fleeting opacities	Peripheral blood eosinophilia and high IgE level Elevated eosinophilic count on bronchoalveolar lavage	1
**H1N1**					PCR test revealed HINI virus	1
**ARDS**		Clinical criteria		Bilateral basal dense consolidation on a background of diffuse GGO in the non-dependent regions with bronchial dilatation in GGO and crazy paving appearance		3
**Hypersensitivity pneumonitis**		Clinical history of exposure to antigen (Bird Fancier’s Disease) Reproduction of symptoms following exposure		Poorly defined centrilobular nodules Headcheese lung in subacute phase	Lymphocytosis on bronchoalveolar lavage	4
**RB-ILD**		History of smoking No fever		Ground-glass opacities and centrilobular nodules	Bronchoalveolar lavage (BAL) findings (the presence of smokers’ macrophages and the absence of lymphocytosis) Proved by biopsy	2
**Amiodarone lung**		History of drug intake		Peripheral patches Pulmonary nodules and septal thickening	Fiberoptic bronchoscopy with BAL and transbronchial biopsy	1
**SLE with diffuse alveolar hemorrhage**		History of SLE, hemoptysis.		Upper lobe predominance, confluent consolidation sparing costophrenic angles and the lung periphery.		1
**Trauma**		History of recent trauma	Pneumothorax			5
**Atypical bacterial infection**	Mycoplasma bronchopneumonia		Pleural effusion	Peribronchial thickening, confined to lobes		1
	Staph-penumonia			GGO peribronchial thickening and pneumatocele		1
	Other bacterial pneumonia			Unilateral lung affection Tree in bud Bronchiectasis		6
**Alveolar sarcoid**		History of sarcoid No fever		Upper lobe affection, peribronchial thickening, mediastinal lymph nodes		1
**Acute Interstitial Pneumonia**		Acute symptoms like ARDS		Bilateral asymmetric confluent GGO with consolidative patches more in the lower lobes	Proved by transbronchial biopsy	1
**Cryptogenic organizing pneumonia**			Pleural effusion	Atoll sign Subpleural sparing	Confirmed by histopathological correlation	2
**Pulmonary alveolar proteinosis**				Asymmetric lung involvement with crazy paving appearance	Confirmed by bronchoalveolar lavage	1
**Metastatic Calcifications with renal failure**		History of renal failure		High-density centrilobular ground glass nodules with superadded infection		1
**Cardiogenic pulmonary edema**		Clinical history	Enlarged cardiac size +/− pericardial effusion, dilated pulmonary trunk, bilateral pleural effusion,	Thickened interlobular septal, peri-lymphatic, and peribronchovascular thickening Perihilar distribution of ground glass opacities (bat wing)		3

## Discussion

COVID-19 pandemic is a severe and easily transmissible disease exploding all around the world. Chest CT scans play an essential role in the initial and early diagnosis of COVID-19 as it can show positive findings before the initial positive RT-PCR. So, it is important to focus on baseline CT findings and radiologists’ capabilities to differentiate between non-COVID and COVID-19 in the first consultation to provide proper isolation and treatment [12].

Bai et al. [13] cited that radiologists were capable of differentiating COVID-19 from other viral pneumonias by chest CT with high specificity and moderate or varying specificity (24–94%) among 7 different readers from the USA and China, but an easy simple understood system is still needed especially in epidemic areas with poor medical resources and expert radiologists. According to previous studies, COVID-19 is more likely to present with some CT image features compared to non-COVID-19 diseases.

All studies indicate that the main CT feature of COVID-19 pneumonia is the presence of ground-glass opacities (GGO), typically with a peripheral and subpleural distribution. The multiple lobes involvement with basal predominance is reported in the majority of cases with COVID-19 [10].

GGO can result from various pathologies of alveolar filling with water, pus, protein, blood, or cells including viral infections, like COVID-19, as well as bacterial infections [1].

In their study, Luo et al. [14] added some negative points to make a hierarchical diagnosis. As the most of the reported COVID-19 cases show affection of more than 2 lobes of the lungs, only one lobe involvement is considered as a negative scoring point and also the single-lobe affection has been reported in some cases of community-acquired pneumonia.

Many things in the lungs look exactly the same, and due to the tens of entities that contribute to GGO and/or consolidation, it is critical to understand the new and sometimes puzzling clinical presentations emerging in the current COVID-19 pandemic and it is important to have at hand concepts of non-COVID-19 conditions that act as “mimics and chameleons” of the COVID-19 pneumonia.

One of the major differentials of COVID-19 pneumonia is the pneumonia from other infectious causes like bacterial origin. Community-acquired pneumonia is usually characterized by an airspace consolidation affecting one segment or lobe, limited by the pleural surfaces. CT may also show ground-glass pattern, centrilobular nodules, bronchial thickening, and/or mucoid impaction. In the absence of superinfection, COVID-19 pneumonia has very different findings, with no centrilobular nodules or mucoid impactions [15].

**Fig. 1:**
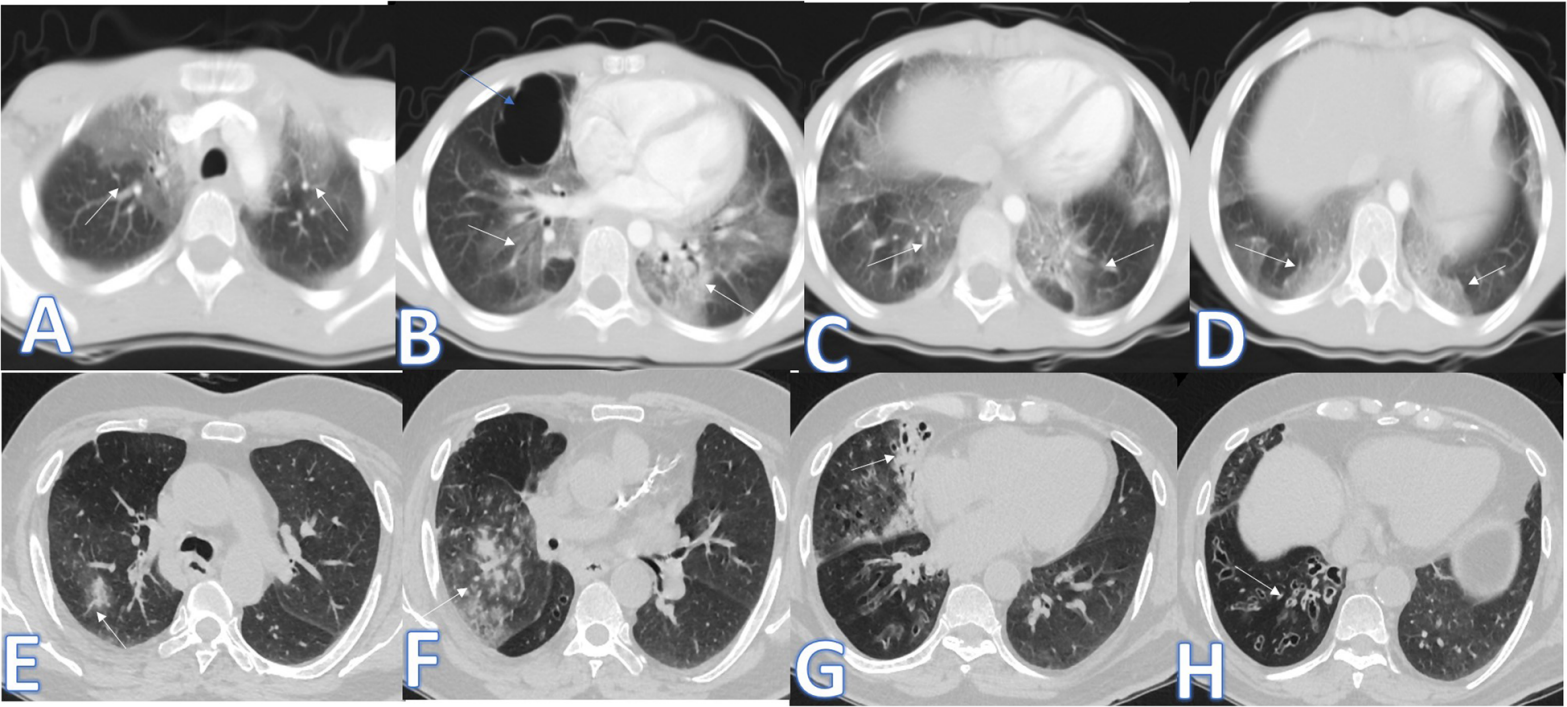
A 10-year-old male patient with fever and cough. CT (**a**–**d**) showed bilateral scattered ground glass patches more central than peripheral (white arrows) with large pneumatocele at the medial segment of the right middle lobe (blue arrow). The diagnosis was staph pneumonia. A 45-year-old male patient with cough and fever. CT (**e**–**h**) showed unilateral right lung consolidative patch with tree-in bud appearance and ground glass appearance (arrows in **e** and **f**) with cylindrical bronchiectasis at the middle and lower lobes (arrows in **g** and **h**) and peribronchial thickening. The diagnosis was bacterial pneumonia

**Fig. 2:**
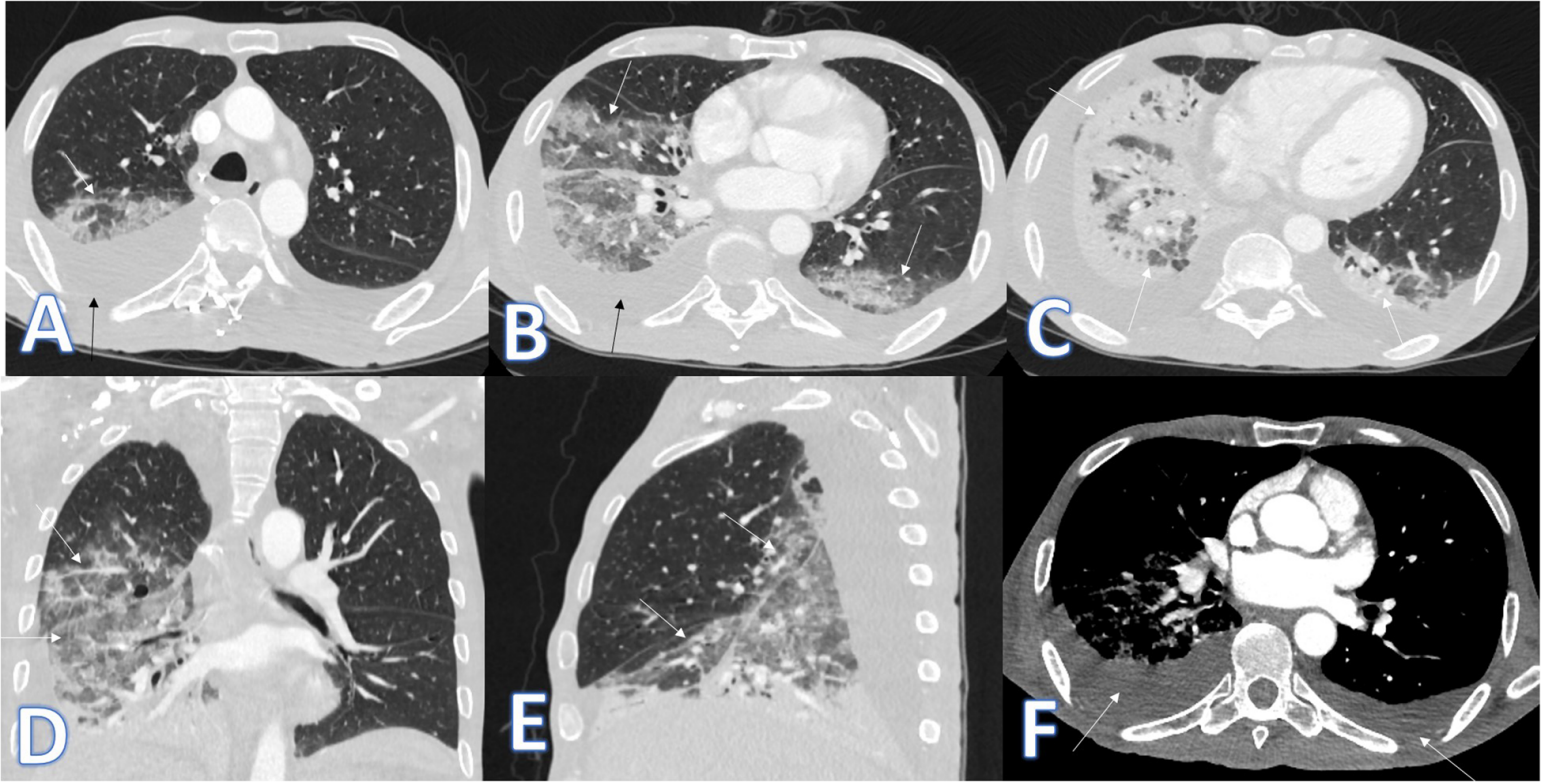
A 53-year-old male patient with cough and dyspnoea. CT showed multiple consolidative patches with ground glass appearance involving the whole right lower lobe, lateral segment of the middle lobe, and superior and posterior segments of the left lower lobe with bilateral pleural effusions (arrows). The diagnosis was mycoplasma pneumoniae

**Fig. 3:**
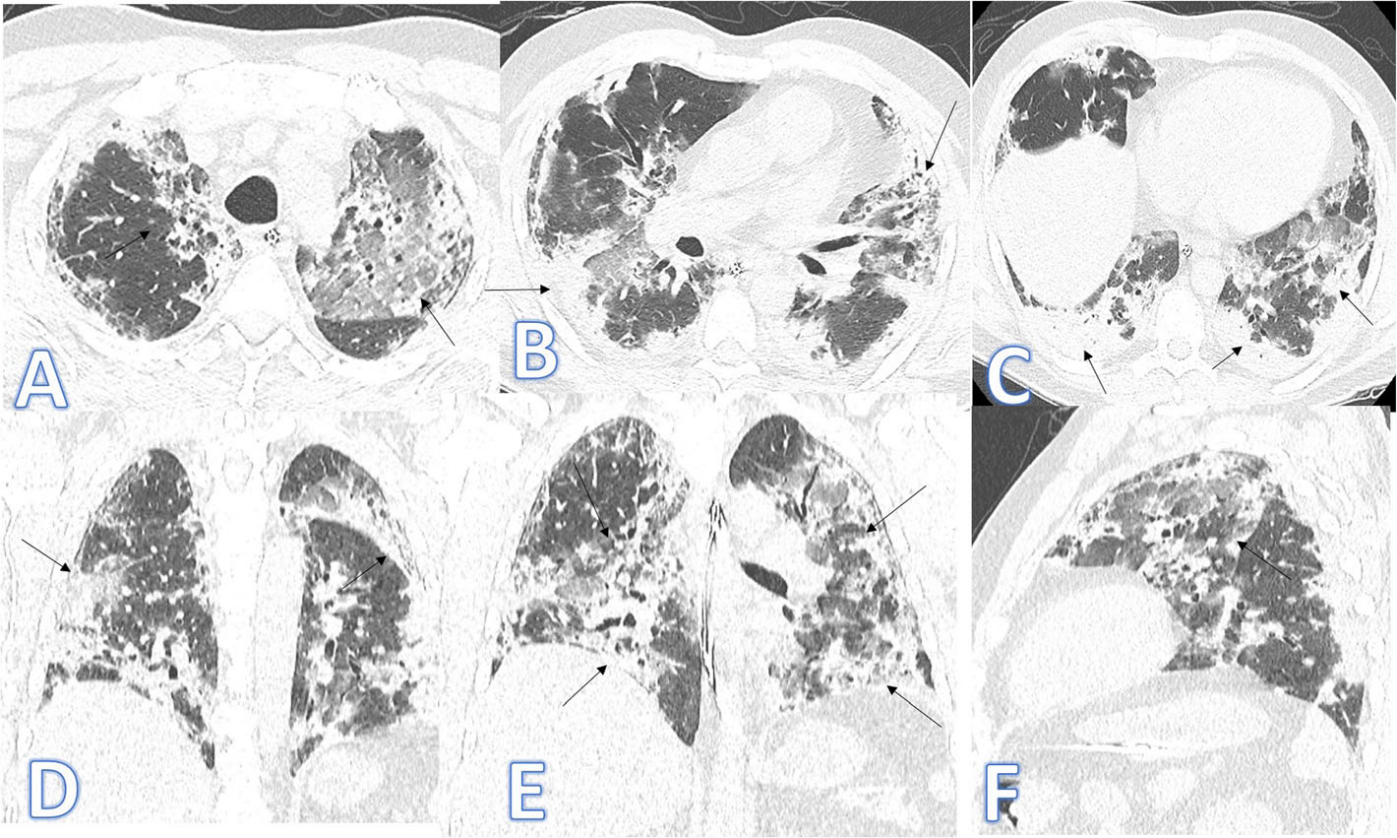
A 59-year-old male with cough, desaturation with fever. CT chest showed bilateral confluent consolidative patches with GGO and crazy paving appearance (arrows), and it was proven H1N1 on PCR test

In this study, there were 8 cases of atypical bacterial pneumonias, and they showed GGO with consolidative patches like COVID-19, but there were other features rarely seen in COVID-19. A large pneumatocele is seen in staph pneumonia (Fig. 1), unilateral lung affection with consolidative patch and tree-in-bud appearance with cylindrical bronchiectasis (Fig. 1). Also, consolidation confined to the lobes with peribronchial thickening and pleural effusion in Mycoplasma pneumoniae (Fig. 2). There was one case of CT feature typical to COVID-19. CT chest showed bilateral confluent consolidative patches with GGO and crazy-paving appearance, and it was proven H1N1 on PCR test (Fig. 3).

Elicker et al. [16] and Grudzinska et al. [17] stated that in the current epidemic, GGO in patients with fever and respiratory symptoms suggests COVID-19 until proved otherwise, but some pneumonias of other viral causes may show some different signs like H1N1 influenza, with bronchial wall thickening, centrilobular nodules, and a distribution more along the bronchovascular bundles. Pleural effusion, pneumothorax/pneumomediastinum may be present.

There are broad spectra of non-infectious conditions that cause diffuse GGO. In this study, there were 3 cases of cardiac pulmonary edema with cardiomegaly, dilated pulmonary trunk, interlobular septal and peribronchovascular thickening, pleural effusions, and perihilar distribution of ground-glass opacities.

Komiya et al. [18] stated that pulmonary edema (cardiogenic and non-cardiogenic) is one of the most common causes of diffuse GGO characterized by central predominance with sparing of the peripheral portions of the lung contrary to COVID-19. It is associated with other suggestive signs such as bronchovascular bundle thickening, interlobular septal thickening, and pleural effusions.

**Fig. 4:**
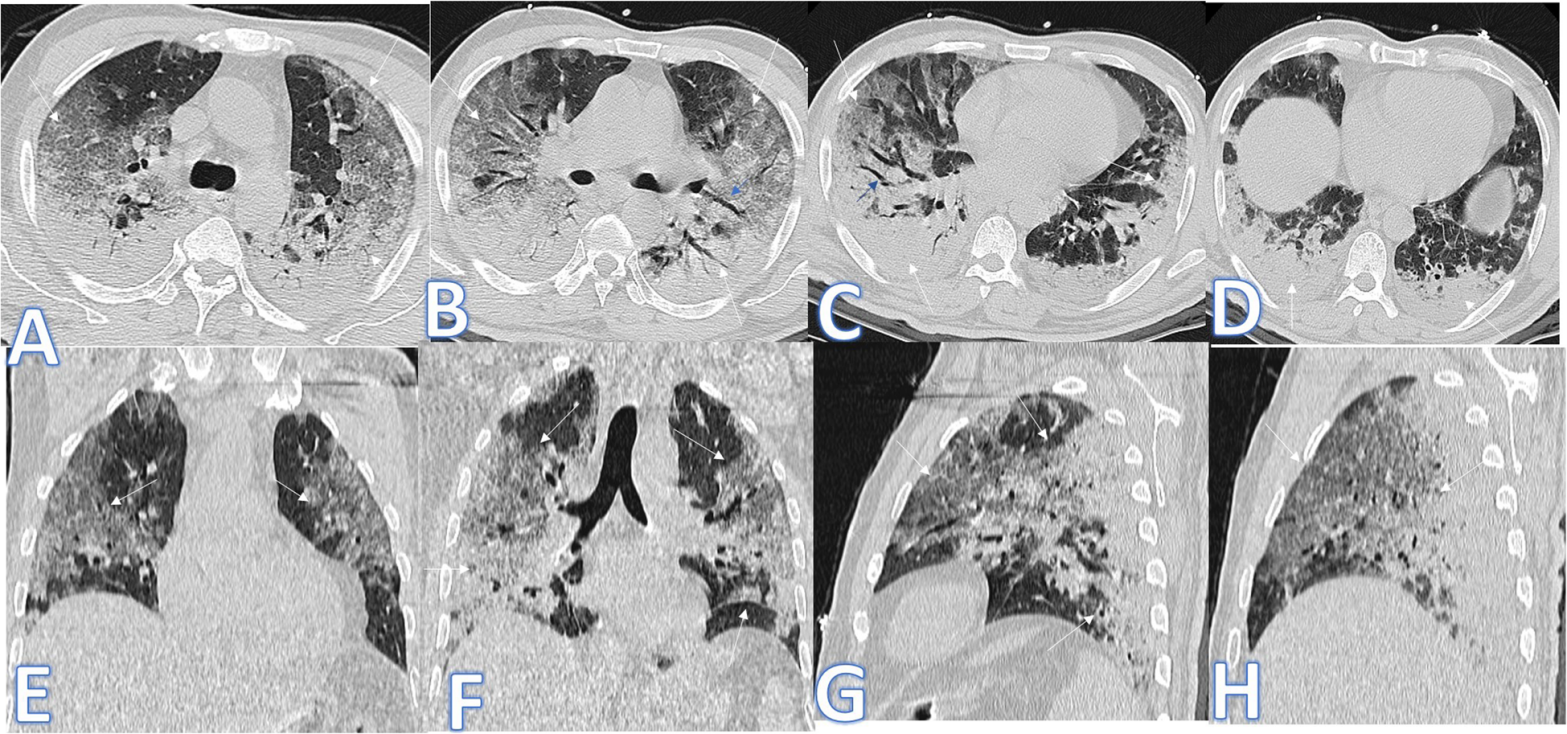
A 68-year-old male patient with septicemia, presented with fever and cough. CT showed bilateral basal dense consolidations on background of GGO in the anterior non-dependent portions of the lungs and crazy paving appearance (white arrows) with bronchial dilatation in the ground glass opacities (small blue arrows) with no pleural effusion, and the diagnosis was ARDS

In this study, there were 3 cases of ARDS, and the diagnosis was based on acute clinical criteria and imaging features of bilateral basal extensive consolidation on the background of GGO in the anterior non-dependent portions of the lungs with bronchial dilatation in the ground glass opacities and crazy-paving appearance (Fig. 4).

Zompatori et al. [19] and Ferguson et al. [20] described acute respiratory distress syndrome (ARDS) diagnosis is based mainly on clinical criteria include lung injury of acute onset, within 1 week of an apparent clinical insult and with the progression of respiratory symptoms, respiratory failure not explained by heart failure or volume overload and decreased arterial PaO_2_/FiO_2_ ratio. According to the phase of the disease, in the early phase, CT imaging shows pulmonary opacification: with anteroposterior density gradient with basal dense consolidation on a background of diffuse GGO with normal or hyperexpanded lung in the non-dependent regions.

**Fig. 5:**
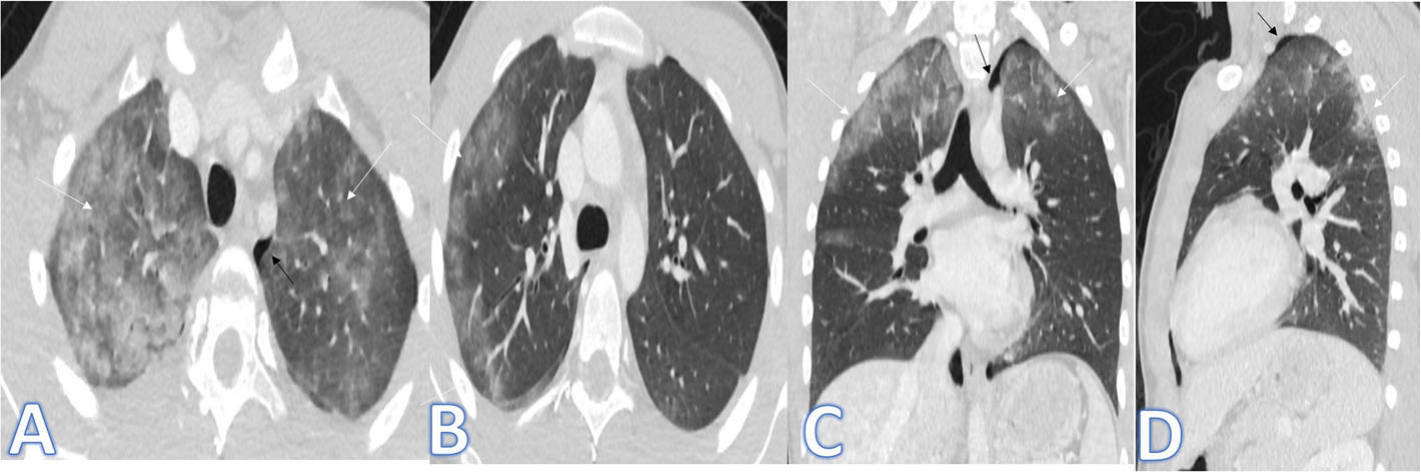
A 19-year-old male patient presented with chest tightness after trauma. He had a history of trauma 1 day ago. CT chest showed bilateral GGO at both the upper lobes and right lower lobes with peripheral predilection (white arrows), the thin rim of the left pneumothorax (black arrow) was seen on the left side, and the diagnosis was post-traumatic pulmonary contusions

In this study, there were 5 cases of pulmonary contusions following trauma, they showed scattered GGO, the clue for the diagnosis was the clinical history with the presence of a thin rim of pneumothorax in 2 cases (Fig. 5).

Oikonomou et al. [21] stated that pulmonary contusions follow blunt or penetrating chest trauma and are almost always seen with other chest (and abdominal) injuries, typically seen as focal, non-segmental areas of parenchymal opacification, more common posteriorly, and in the lower lobes usually peripheral. The clinical history of trauma is always a distinguishing feature.

Wallis et al*.* [22] stated that some of interstitial lung disease (ILD) also involve the alveolar spaces that make an overlap with COVID-19 pneumonia. This includes COP, HP, RB-ILD, sarcoid, PAP, vasculitis, rheumatoid disease, and drug-induced.

**Fig. 6:**
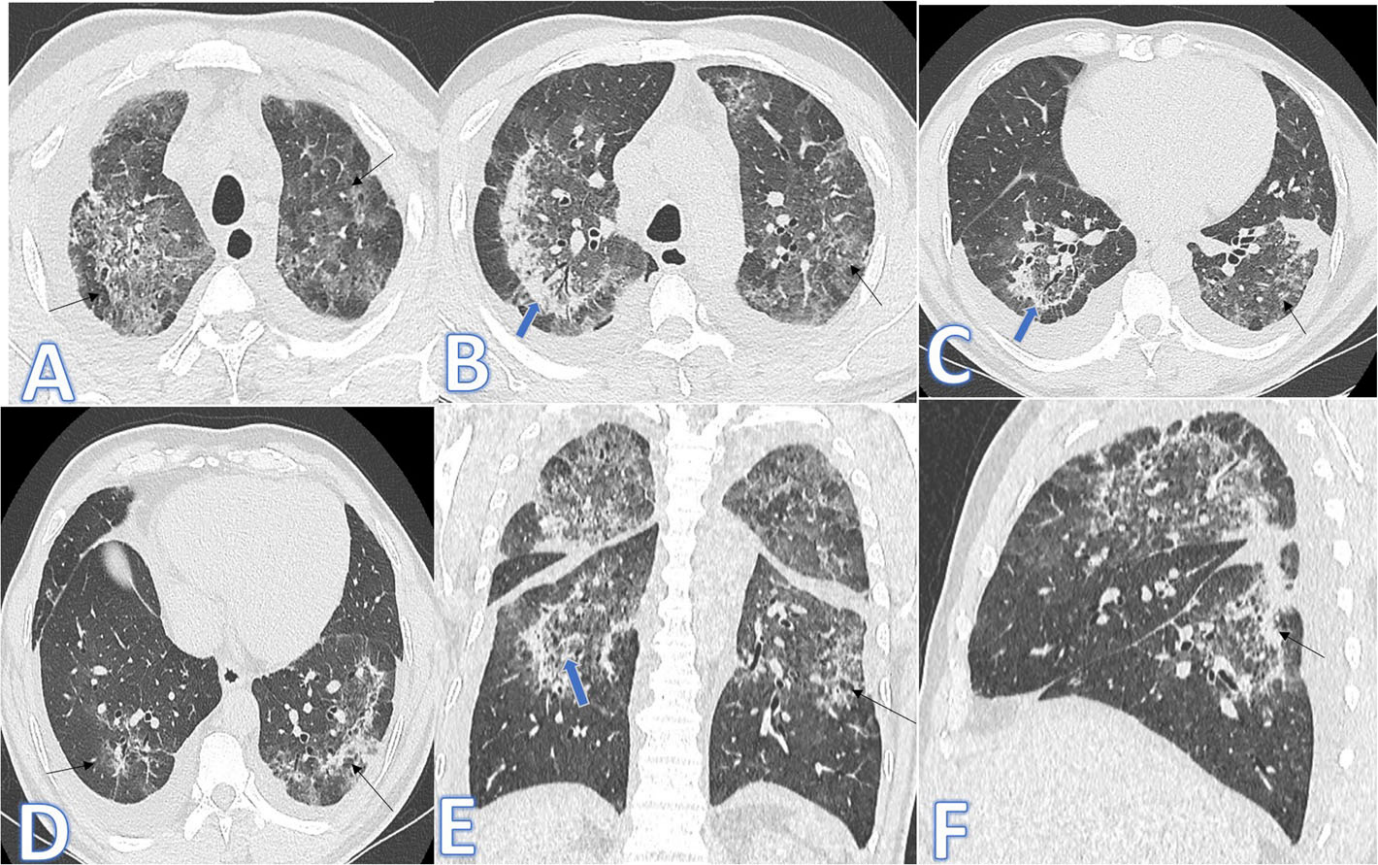
A 61-year-old male patient presented with cough and fever. CT chest showed multifocal GGO, crazy paving and consolidation with Atoll sign (blue arrows) more in the upper lobes with subpleural sparing and bilateral pleural effusions more on the right side (arrow in **d**). The diagnosis was COP, confirmed by histopathology

In this study, there were 2 cases of cryptogenic organizing pneumonia with multifocal GGO, crazy-paving and consolidation with atoll sign more in the upper lobes and bilateral pleural effusions (Fig. 6). They were mimickers to COVID-19 pneumonia with histopathological results that were helpful for the final diagnosis.

Webb et al*.* [23] described that the most common HRCT features of cryptogenic organizing pneumonia (COP) with multifocal ground glass opacifications, crazy-paving and/or consolidation, small, ill-defined nodules, bronchial wall thickening, or dilatation. He stated that the reverse halo sign (atoll sign) is seen only in 20% of patients, and it is not considered to be highly specific.

**Fig. 7:**
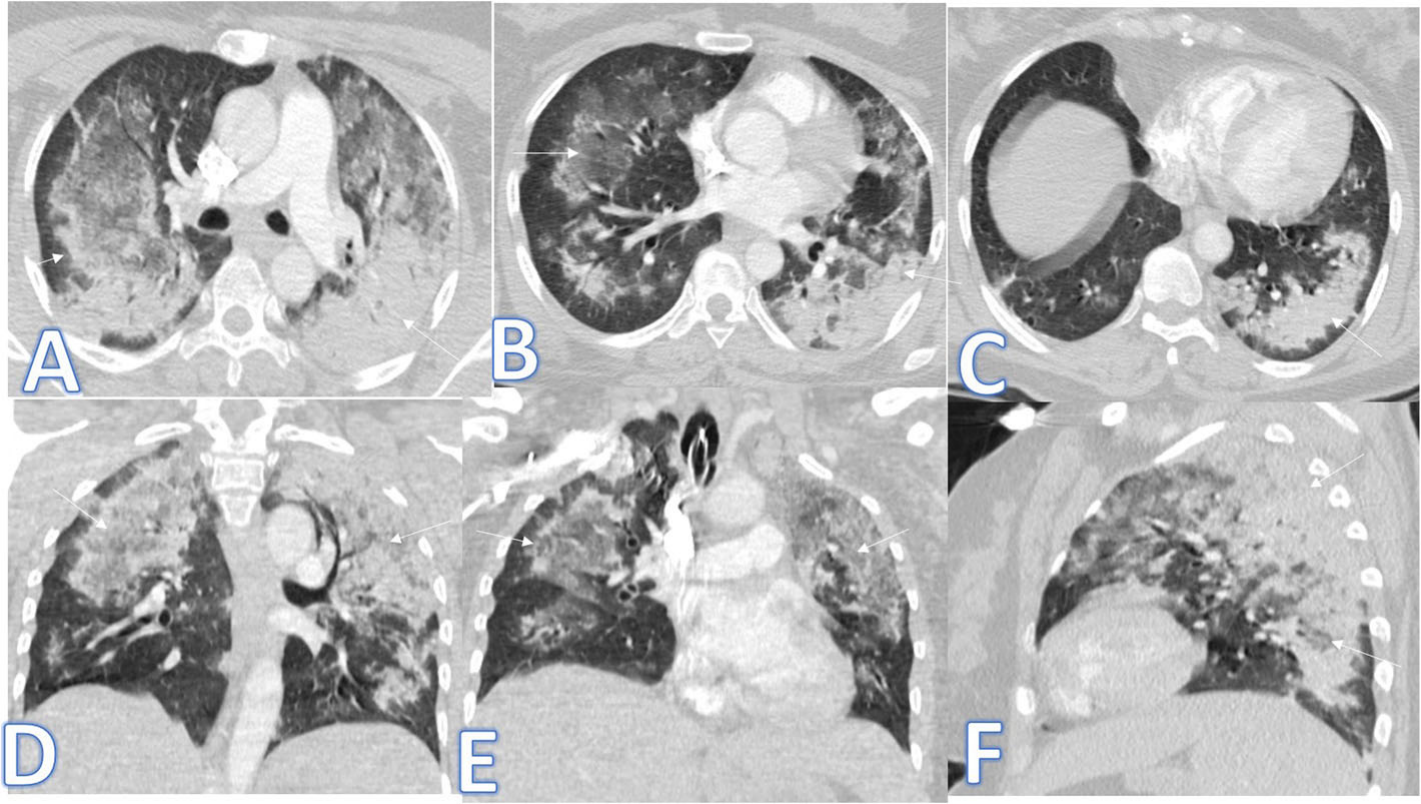
A 46-year-old female patient with SLE, presented with hemoptysis. CT showed bilateral confluent consolidative patches with GGO (arrows) more predominant in the upper lobes with subpleural and costophrenic angle sparing. The diagnosis was diffuse alveolar hemorrhage

In the current study, there was one case of diffuse alveolar hemorrhage (Fig. 7) with a patient known of SLE and presented with hemoptysis. The predominance of GGO in the upper lobes with subpleural and costophrenic angle sparing as well as the clinical history was the diagnostic clues.

Marten et al. [24] describe intra-alveolar hemorrhage secondary to extensive parenchymal small-vessel vasculitis like that caused by connective tissue disorders like systemic lupus erythematosus (SLE) which is typically diffuse and initially causes more widespread lobular ground-glass opacification with gravity-dependent density progressing to air-space consolidation +/− crazy-paving pattern.

**Fig. 8:**
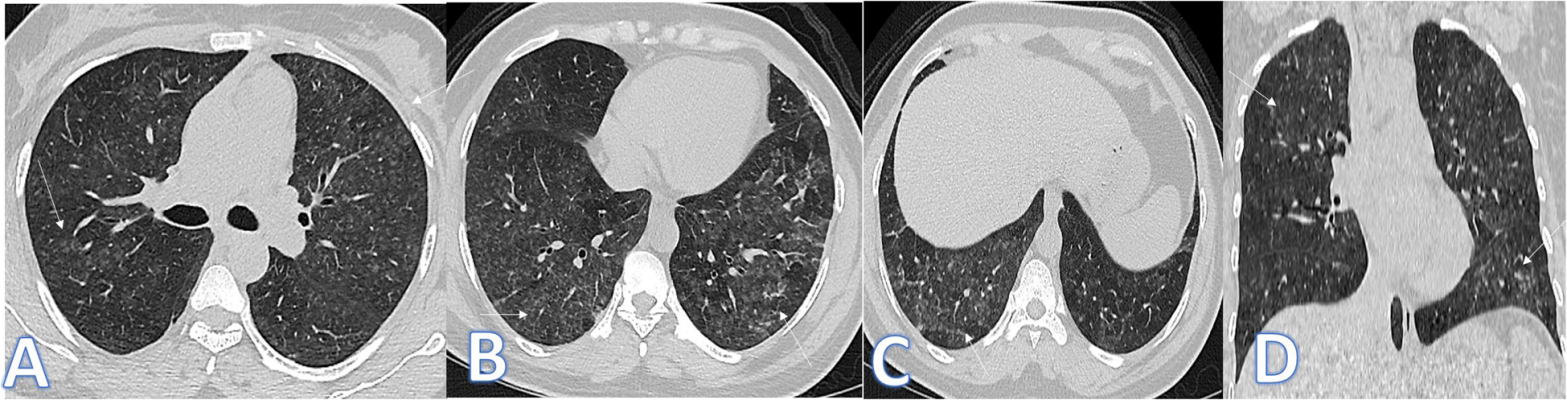
A 46-year-old female patient with acute HP presented with fever and cough. CT chest showed bilateral scattered centrilobular nodules with bilateral lower lobe GGO (arrows). No fibrosis seen

**Fig. 9:**
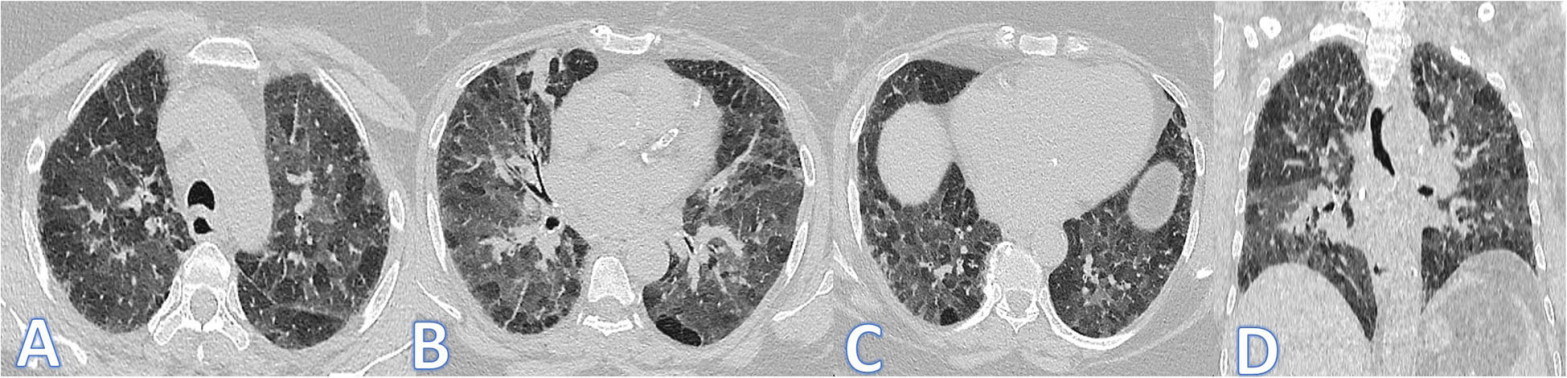
A 64-year-old female axial and coronal CT image shows the headcheese sign, featuring sharp geographic margination along the edges of the secondary pulmonary lobules with three distinct levels of attenuation representing normal, ground glass, and hyperinflated regions of lung. The diagnosis was subacute HP

In this study, there were 4 cases of hypersensitivity pneumonitis of both acute (Fig. 8) and subacute phases (Fig. 9). The diagnosis was made by the clinical history of exposure to antigen (Bird Fancier’s Disease), radiological findings with small poorly defined centrilobular nodules, ground-glass appearance in the acute phase or headcheese sign in the subacute phase, lymphocytosis on bronchoalveolar lavage, and reproduction of symptoms following exposure.

Lacasse et al. [25] described that acute hypersensitivity pneumonitis or acute extrinsic allergic alveolitis is usually occurring within few hours after antigen exposure and often recurs with the re-exposure and has the potential to resolve with treatment. In the acute phase, chest CT shows bilateral and symmetric homogeneous GGO (alveolitis) with multiple centrilobular opacities: usually < 5 mm in diameter also may be present with no fibrosis [26].

**Fig. 10:**
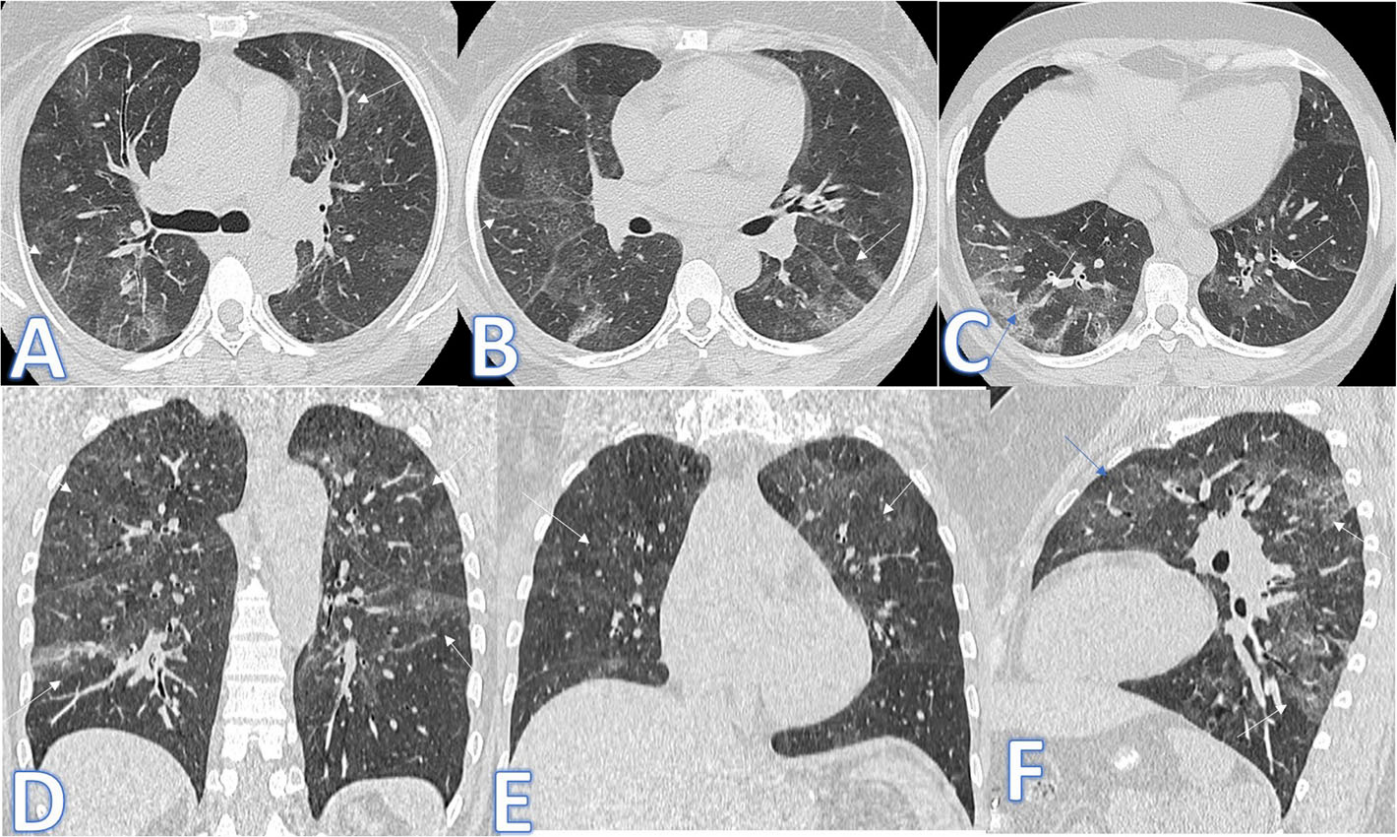
A 45-year-old male patient presented with cough and dyspnea. CT showed bilateral patches of GGO in both lobes (white arrows) with crazy paving seen in both lower lobes (blue arrow); the diagnosis was RB-ILD proven by biopsy

There were two cases of RB-ILD in this study presented by cough, dyspnea, and shortness of breath. Both were heavy smokers. The diagnosis was made by clinical history of smoking, typical HRCT findings of ground-glass opacities, and centrilobular nodules (Fig. 10) and proved by biopsy in the first case and bronchoalveolar lavage (BAL) findings (the presence of smokers’ macrophages and the absence of lymphocytosis) in the second case.

Mavridou et al. [27] described CT findings of RB-ILD with smoking bronchial wall thickening and centrilobular emphysema in addition to ground-glass opacities with the slight upper zone predominance and ill-defined centrilobular nodules.

**Fig. 11:**
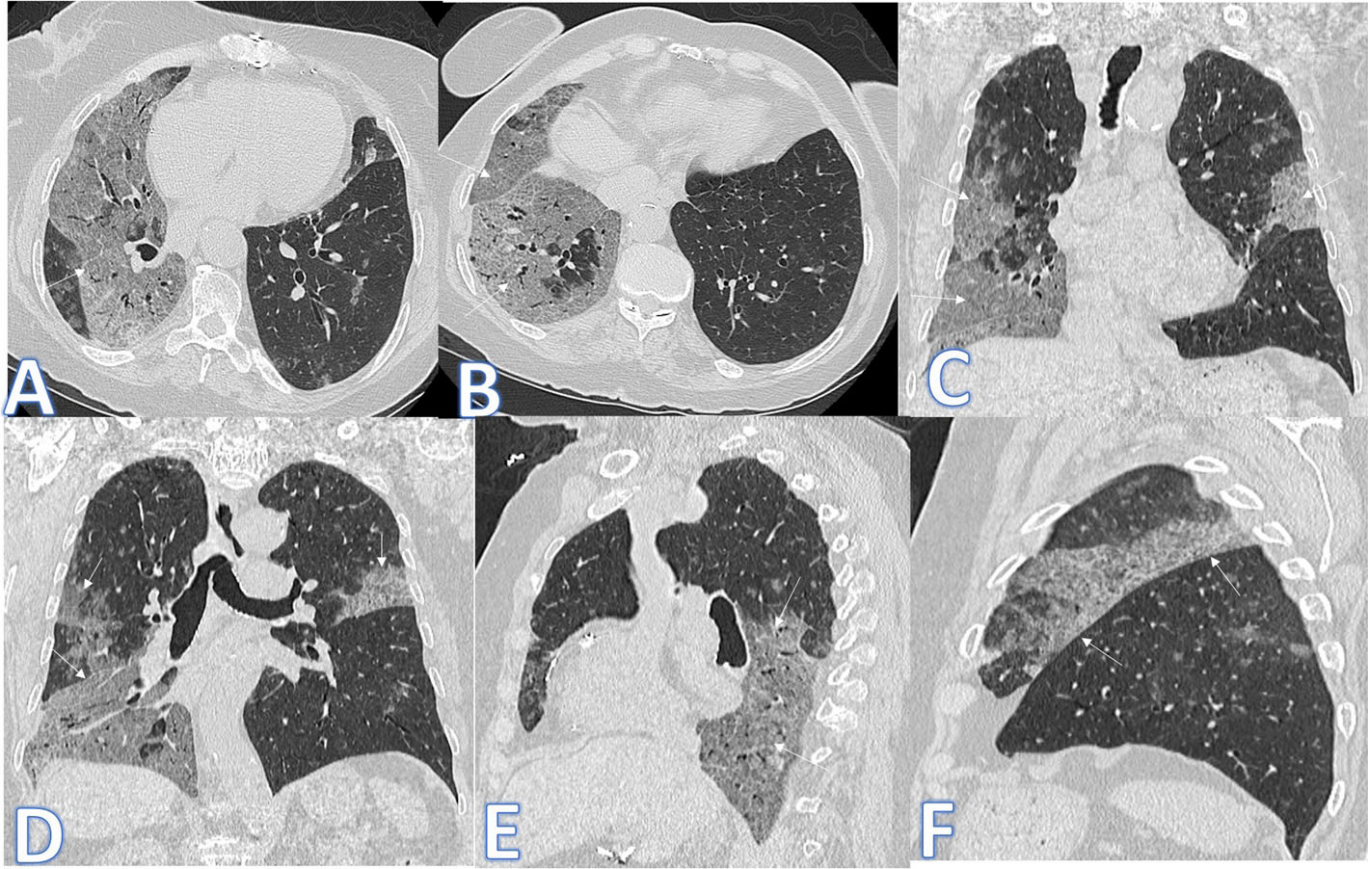
A 80-year-old female patient presented with cough. CT showed bilateral asymmetrical GGO with crazy paving appearance (arrows) near totally involving the right middle and lower lobes with less involvement of the left lung. The diagnosis was PAP

In this study, there was one case of pulmonary alveolar proteinosis (Fig. 11). It mimics CT features of COVID-19 pneumonia with bilateral asymmetrical GGO with crazy-paving appearance, and the diagnosis was confirmed by bronchoalveolar lavage.

Holbert et al. [28] stated that crazy-paving pattern of PAP on CT is non-specific occurring in other diseases.

**Fig. 12:**
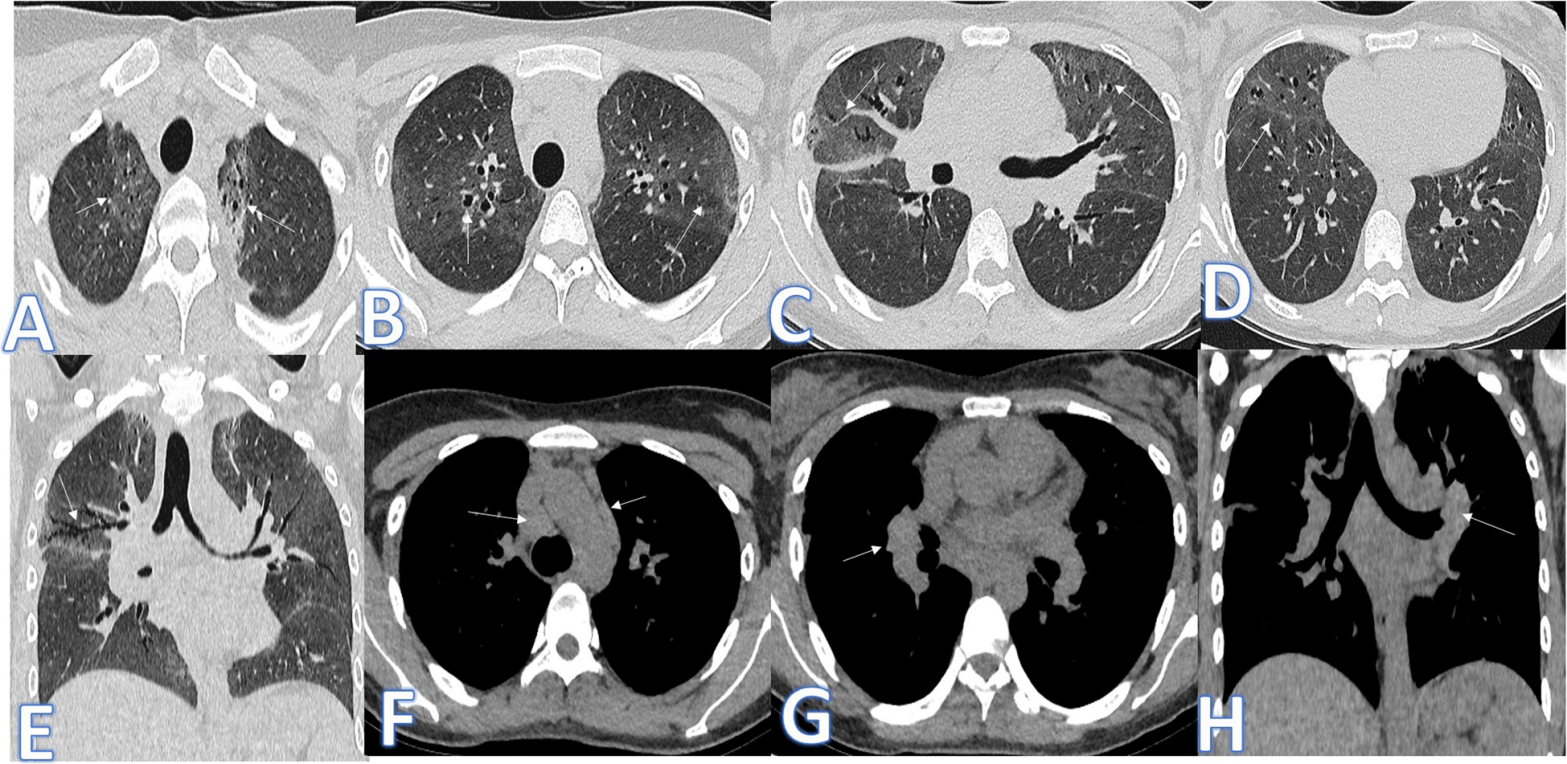
A 30-year-old female patient known of sarcoid disease presented with cough; there was bilateral patchy ground glass appearance with mild bronchiectatic changes (arrows) and fibrotic changes of the upper lobe predilection. Multiple enlarged prevascular, pre/paratracheal, and hilar LNS was seen in mediastinal window (arrows in **f**–**h**)

In this study, there was one case of known sarcoidosis presented with cough. CT showed bilateral patchy ground-glass appearance, but the presence of mild bronchiectatic changes of upper lobe predilection, multiple enlarged prevascular, pre/paratracheal, and hilar LNS as well as the clinical history with no fever was the clue for the diagnosis of alveolar sarcoidosis (Fig. 12).

**Fig. 13:**
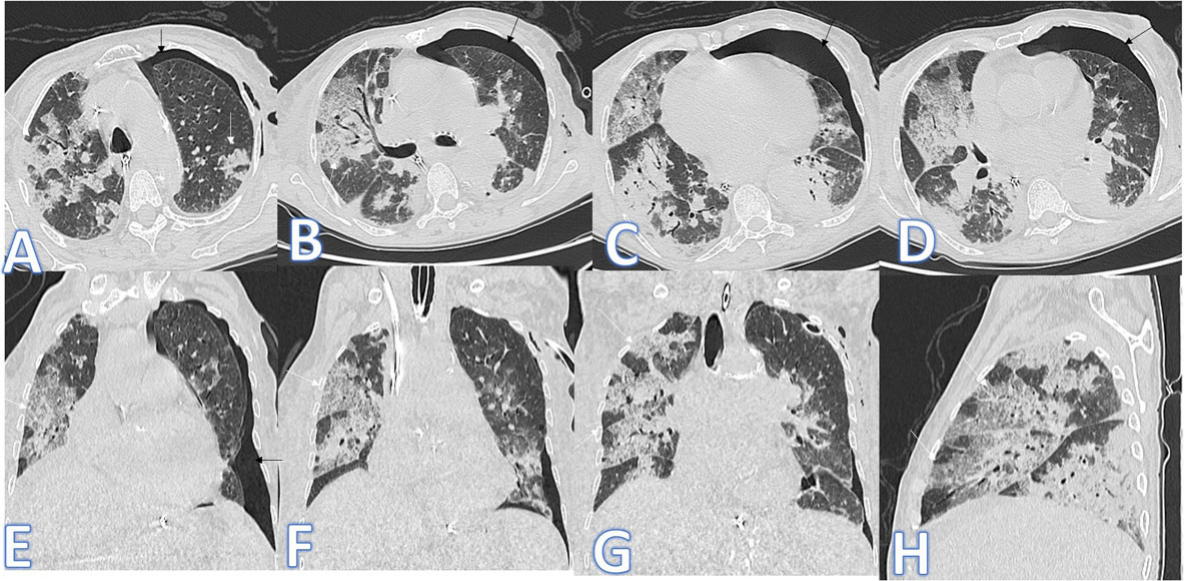
A 72-year-old female patient presented with severe dyspnea with desaturation. She was intubated. CT chest revealed bilateral symmetric confluent patches of ground glass with crazy paving appearance mixed with consolidative patches with air bronchogram more in the lower lobes (white arrows) more on the right side. LT side mild pneumothorax was noted (black arrows). It was diagnosed as AIP after transbronchial biopsy

There was one case of acute interstitial pneumonitis (AIP) presented with severe dyspnea and desaturation; she was intubated. CT chest revealed bilateral asymmetric confluent patches of ground glass with crazy-paving appearance mixed with consolidative patches with air bronchogram more in the lower lobes. The presence of LT side mild pneumothorax was uncommon for COVID-19 pneumonia (Fig. 13). Bronchoalveolar lavage and transbronchial biopsy were done after exclusion of infectious cause.

Wittram et al. [29] described AIP or Hamman-Rich syndrome CT findings with ground-glass attenuation: generally tend to be bilateral and symmetrical, traction bronchiectasis (80% of cases during the course of the disease ), and parenchymal architectural distortion of the lung.

**Fig. 14:**
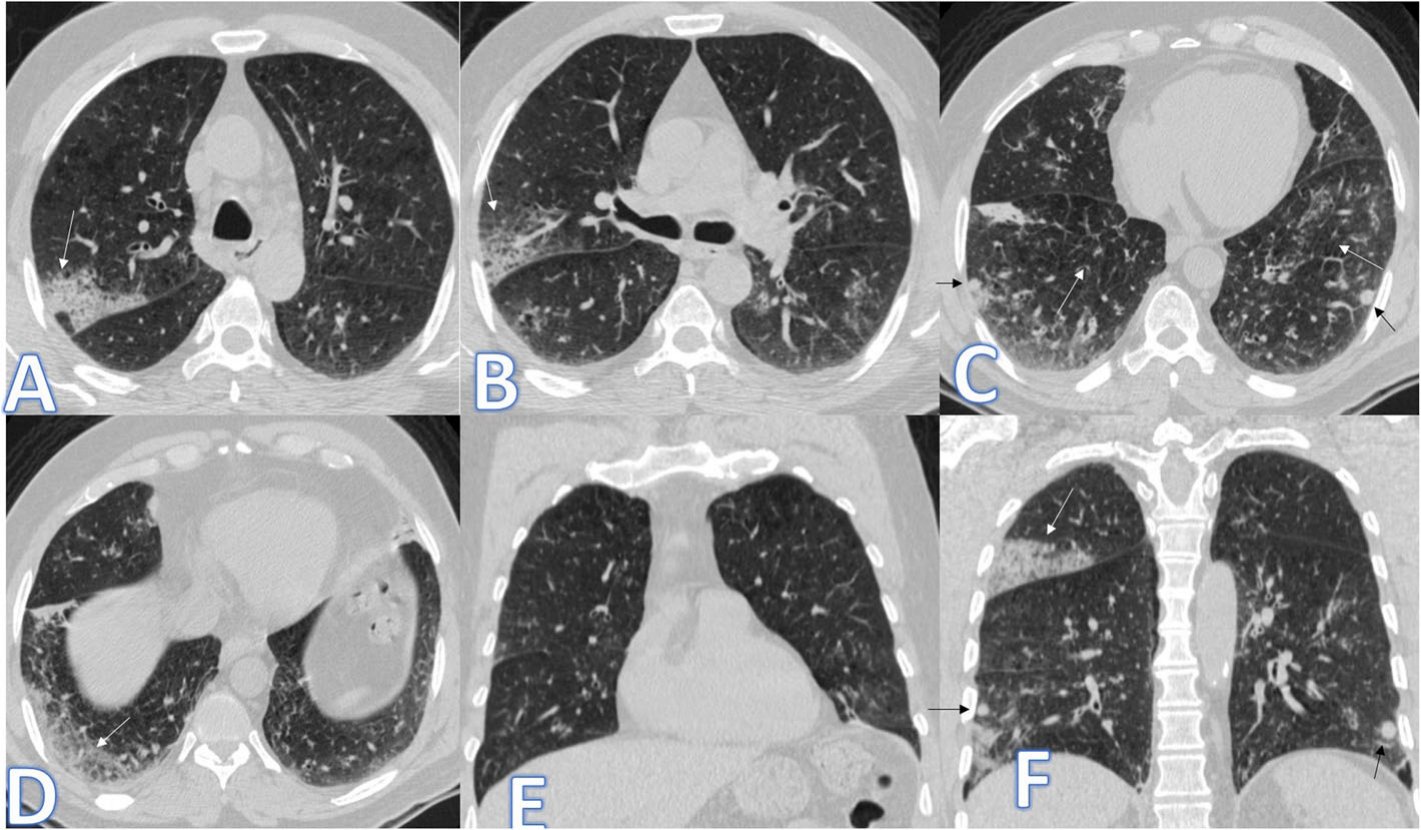
A 65-year-old male patient presented with dyspnea and low-grade fever. CT chest showed bilateral scattered mainly peripheral consolidative patches with faint GGO (white arrows), associated bilateral basal interlobular septal thickening with small nodules (black arrows). The diagnosis was amidarone lung toxicity

There was one patient in this study, presented with dyspnea and low-grade fever with CT chest showing bilateral scattered mainly peripheral consolidative patches with faint GGO, associated bilateral basal interlobular septal thickening with small nodules (Fig. 14). The patient had a history of amiodarone drug intake for 3 years. The patient was COVID-19 negative, and the presence of the pulmonary nodules and interlobular septal thickening was rare features of COVID-19. The case was confirmed by fiberoptic bronchoscopy with BAL and transbronchial biopsy to be amiodarone lung toxicity.

Wolkove et al. [30] stated that amiodarone lung toxicity is more common in patients exposed to amiodarone, usually for at least 6 months with risk factors like age over 60 years and daily dose > 400 mg. It has two main patterns, multiple peripheral GGO and interstitial fibrosis. One of the radiographic appearances of amiodarone pulmonary toxicity is the presence of peripherally located single or multiple pulmonary nodules, or mass-like opacities and may abut the pleura due to localized accumulation of the drug in an area of previous inflammation.

**Fig. 15:**
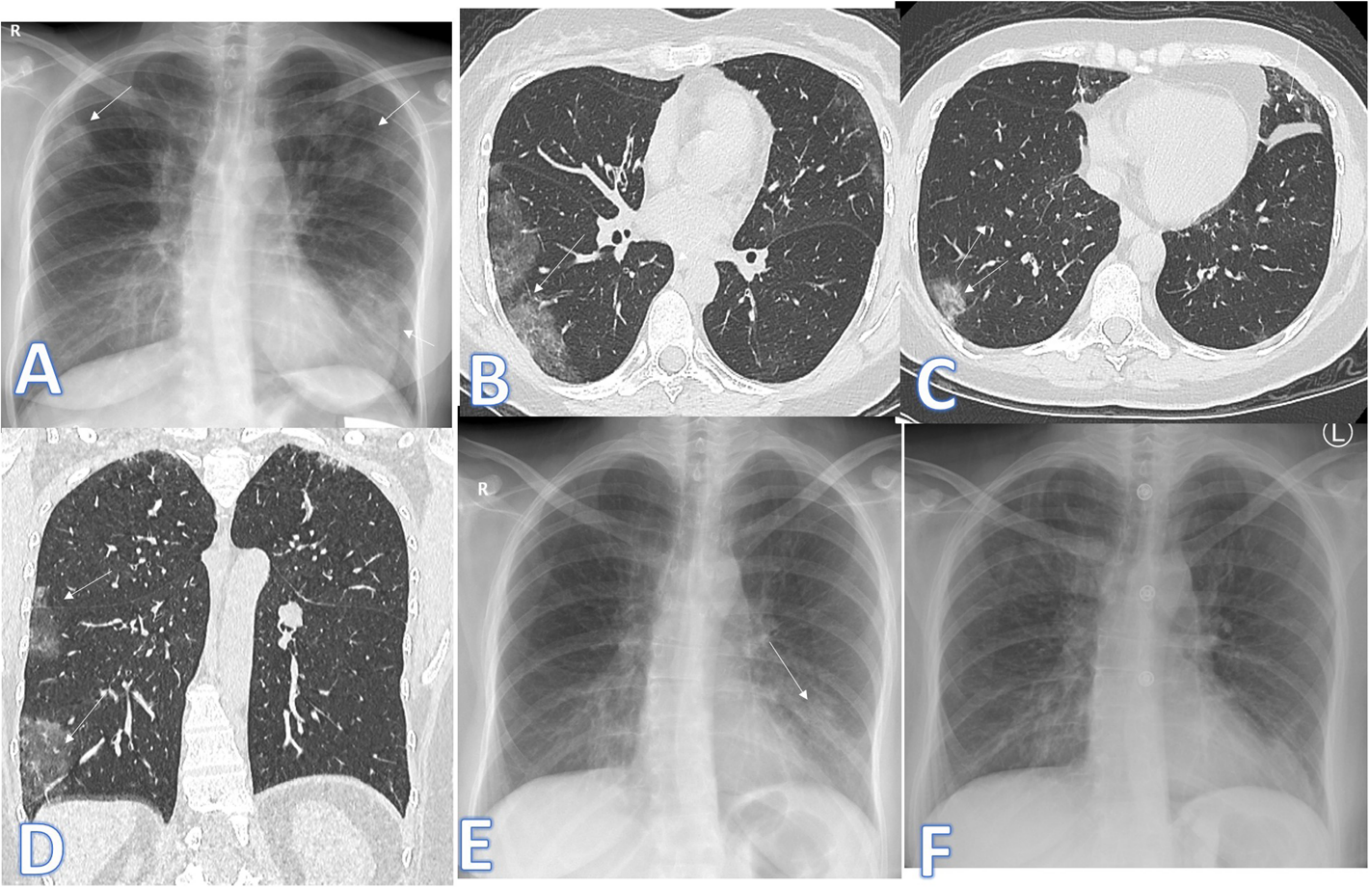
A 38-year-old female patient with fever and cough. Initial X-ray (**a**) showed bilateral small air space opacities (arrows). On CT chest, there was bilateral peripheral GGO (arrows). X-ray done 1 year before (**e**), and there was a left lower lobe air space opacity which was resolved on X-ray (**f**) 6 months later (fleeting). The diagnosis was Loffler’s pneumonia

Another entity of diseases that may mimic COVID-19 is eosinophilic lung disease; we had 3 cases of the eosinophilic lung. The first case was Loffler’s pneumonia (Fig. 15). The diagnosis was made due to fleeting opacities (on X-ray done 1 year before the clinical symptoms, there was left lower lobe air space opacity which was resolved on X-ray 6 months later indicating fleeting opacities), elevated eosinophilic count in BAL, peripheral blood eosinophilia, and high IgE level.

Jeong et al. [31] described Löffler syndrome or simple pulmonary eosinophilia on imaging as a fleeting, non-segmental GGO which may be unilateral or bilateral peripheral predominance. Pleural effusions and lymphadenopathy are not features.

**Fig. 16:**
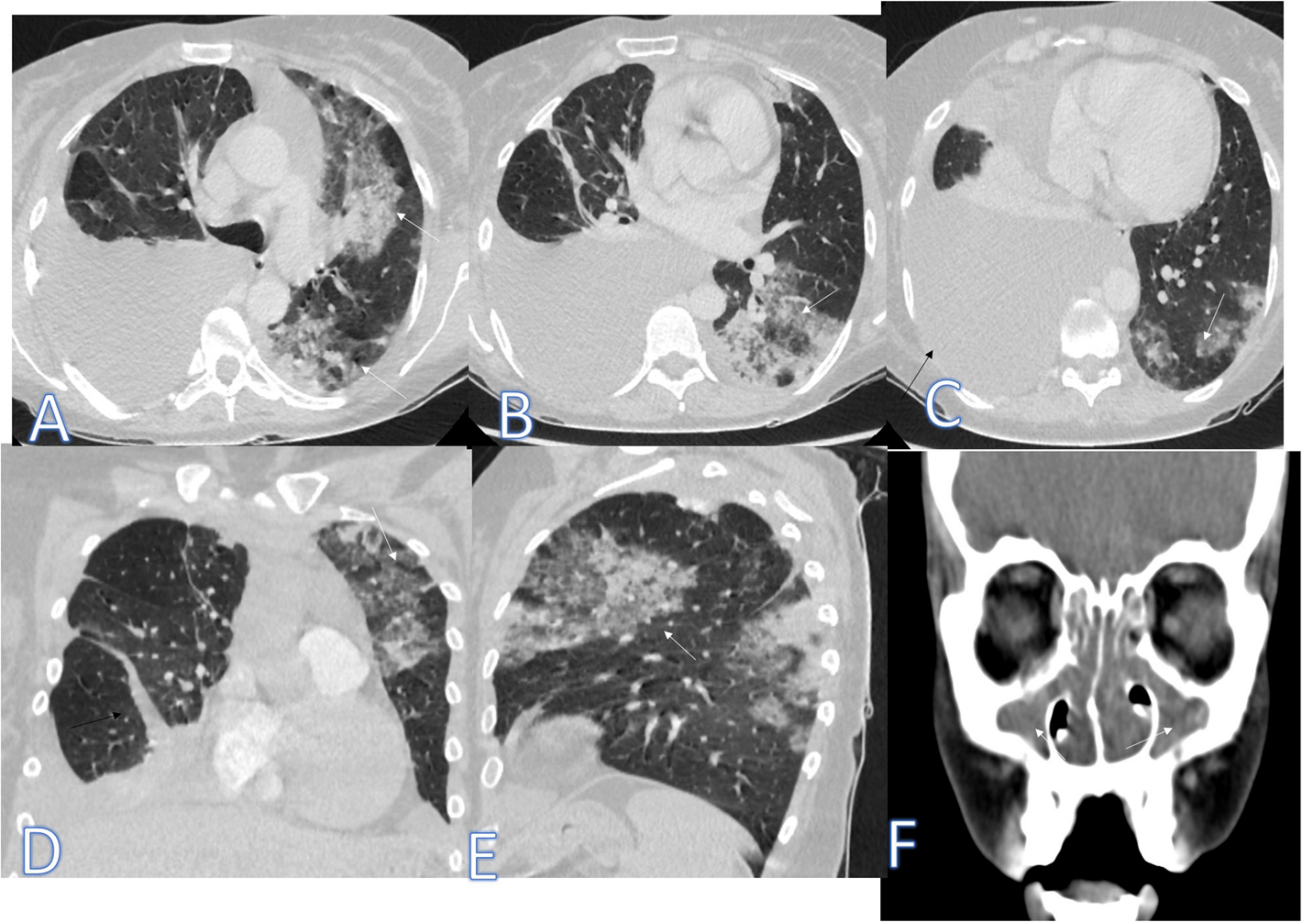
A 39-year-old asthmatic female patient presented with shortness of breath with cough and dyspnea. CT showed multiple consolidative patches with GGO and crazy paving appearance (white arrows) and moderate right pleural effusion (black arrow). CT paranasal sinus (**f**) showed bilateral total opacifications of the sinus (arrow). The diagnosis was Churg-Strauss syndrome

The second case of the eosinophilic lung was Churg-Strauss syndrome (Fig. 16) of asthmatic female patient presented with shortness of breath with cough and dyspnea. CT showed multiple consolidative patches with GGO and crazy-paving appearance, moderate right pleural effusion with extrapulmonary manifestations of pansinusitis, and arthritis, and it was confirmed by laboratory results of high eosinophils and IgE.

The etiology of Churg-Strauss syndrome may be allergic or immune pathogenesis for the disease with asthma, eosinophilia, and elevated serum IgE levels. It affects the lung followed by the skin. However, any organ can be involved. The most common thin-section CT findings include sub-pleural ground-glass opacity or consolidation with a lobular distribution, centrilobular nodules, bronchial wall thickening, and interlobular septal thickening and less commonly mediastinal or hilar lymphadenopathy, and pleural or pericardial effusion [31].

**Fig. 17:**
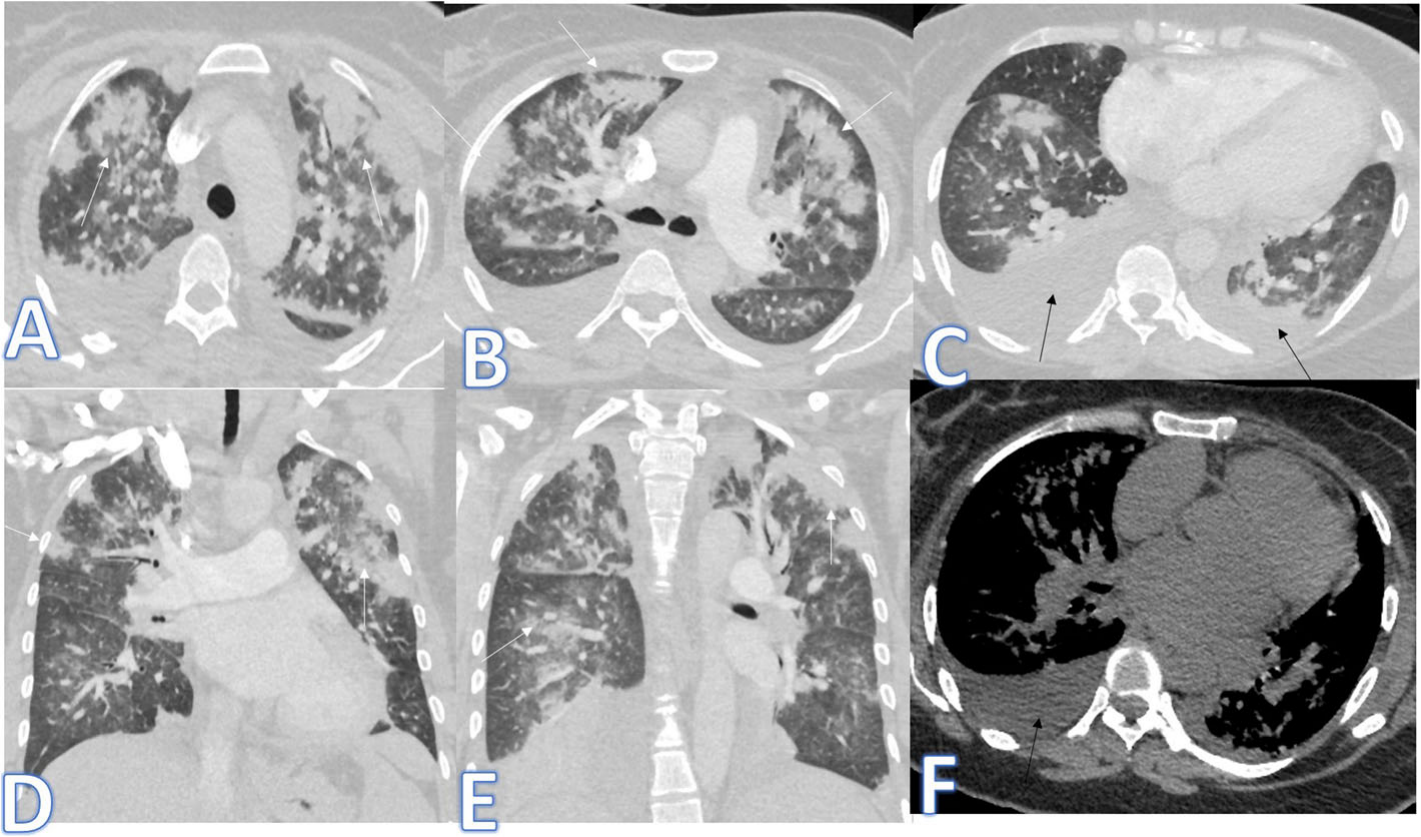
A 49-year-old female patient presented with fever, cough, and dyspnea. CT showed bilateral confluent consolidative patches with upper lobe predilection (white arrows) and bilateral mild pleural effusions (black arrows); the diagnosis was drug rash with eosinophilia and systemic symptoms (DRESS)

The third case of the eosinophilic lung (Fig. 17) was drug rash with eosinophilia and systemic symptoms (DRESS) with a history of drug intake (anticonvulsant), skin rash, fever, cough, and dyspnea. Bilateral confluent consolidative patches with upper lobe predilection, bilateral mild pleural effusions were seen on CT, and the diagnosis was confirmed by peripheral eosinophilia.

The drug rash with eosinophilia and systemic symptoms or DRESS syndrome typically manifests as a skin rash, fever, and lymphadenopathy with variable internal organ involvement and represents a drug-induced hypersensitivity reaction. Chest CT findings are non-specific but may show diffuse multifocal infiltrative opacification [32].

**Fig. 18:**
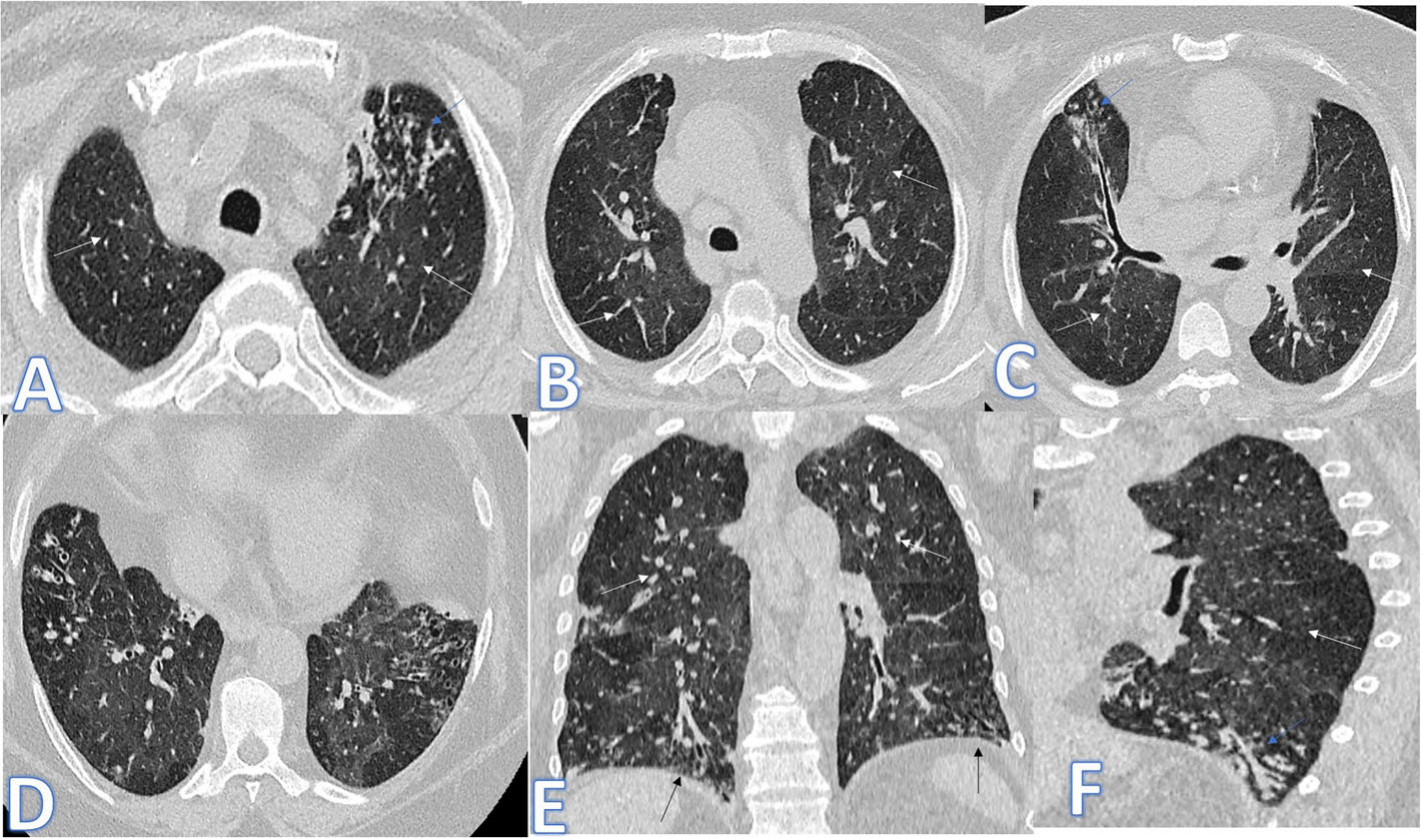
A 61-year-old female patient with bronchial asthma presented with cough and wheezy chest. On CT, there were bilateral mosaic patches of GGO due to air trapping (white arrows) with left upper lobe tree-in bud and subsegmental atelectasis (blue arrows), peribronchial thickening, and bilateral mild basal bronchoectatic changes (black arrows)

There were 3 cases of bronchial asthma in this study (Fig. 18) presented with bilateral mosaic patches of GGO due to air trapping seen, but there was left upper lobe tree-in-bud, subsegmental atelectasis (due to superadded infection), and bilateral mild basal bronchoectatic changes; such features are uncommon for COVID-19.

**Fig. 19:**
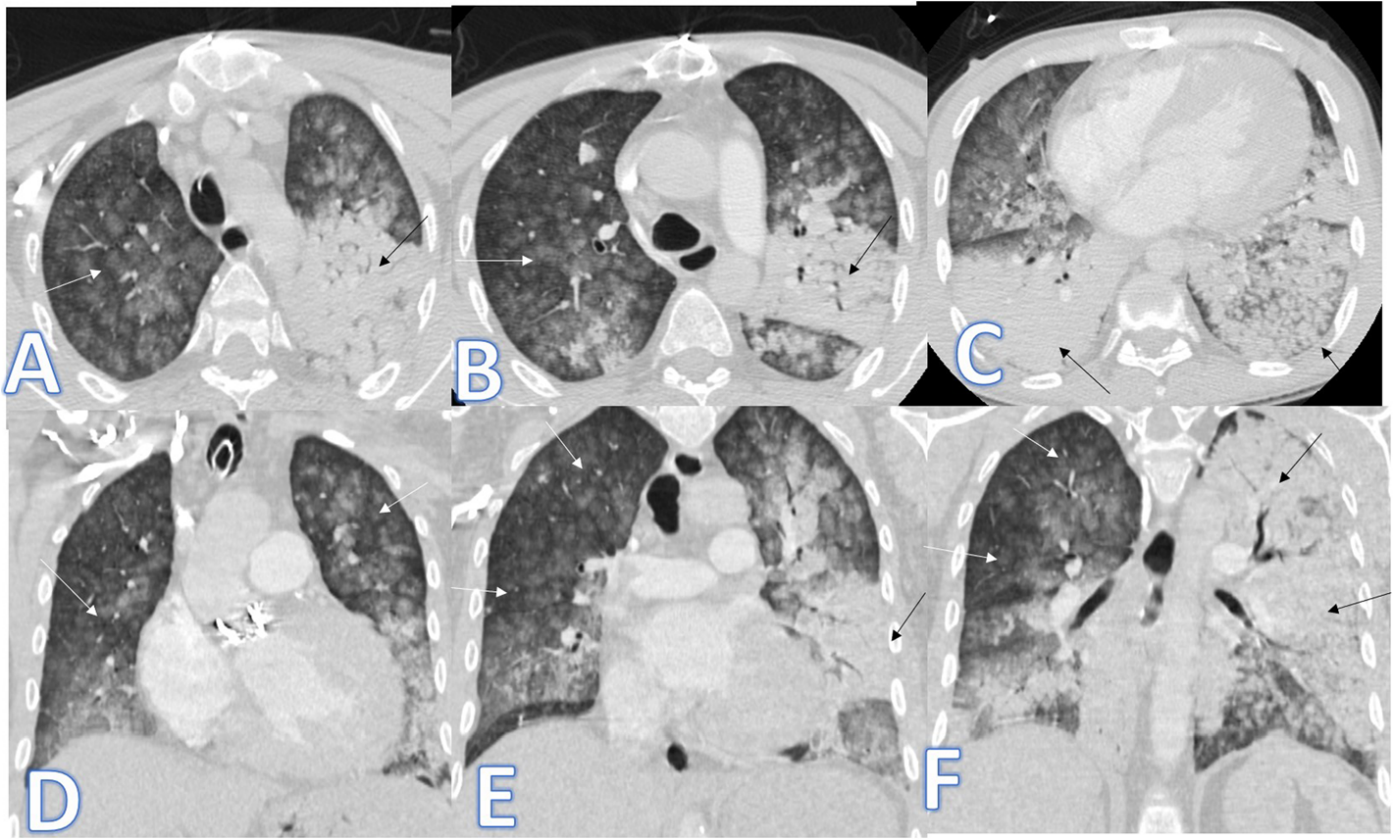
A 32-year-old male patient with ESRD on dialysis presented with fever and cough. CT showed bilateral high-density ground glass centrilobular nodules (white arrows) due to metastatic pulmonary calcification occurring in renal failure. Associated left lung consolidative patches with air bronchogram (black arrows). The diagnosis was metastatic calcification with superadded lung infection

In this study, there was a patient with ESRD on dialysis presented with fever and cough. CT showed bilateral high-density ground-glass centrilobular nodules due to metastatic pulmonary calcification occurring in renal failure. Associated left lung consolidative patches with air bronchogram (Fig. 19). The diagnosis was metastatic calcification with superadded bacterial lung infection based on clinical history with laboratory testing.

## Conclusion

The spectrum of CT imaging findings in COVID-19 pneumonia is wide that could be contributed by many other diseases making the interpretation of chest CTs nowadays challenging to differentiate between different diseases having the same signs and act as deceiving simulators in the era of COVID-19.

## Data Availability

All data and materials are available.

## Abbreviation

nCoV: Novel coronavirus
RT-PCR: Real-time polymerase chain reaction
CT: Computed tomography
NPS: Nasopharyngeal swab
GGO: Ground glass opacification
UIP: Usual interstitial pneumonia
NSIP: Non-specific interstitial pneumonia
ARDS: Acute respiratory distress syndrome
DAD: Diffuse alveolar damage
COP: Cryptogenic organizing pneumonia
SLE: Systemic lupus erythematosus
HRCT: High-resolution computed tomography
ILD: Interstitial lung disease
RB-ILD: Respiratory bronchiolitis interstitial lung disease
PAP: Pulmonary alveolar proteinosis
HP: Hypersensitivity pneumonitis
CHP: Chronic hypersensitivity pneumonitis
BAL: Bronchoalveolar lavage
AIP: Acute interstitial pneumonia
PET: Positron emission tomography
DRESS: Drug rash with eosinophilia and systemic symptoms
ESRD: End-stage renal disease

## References

[1] Song F, Shi N, Shan F (2020). Emerging 2019 novel coronavirus (2019-nCoV) Pneumonia. Radiology.

[2] WHO Director-General’s opening remarks at the media briefing on COVID-19-11 March 2020. https://www.who.int/dg/speeches/detail/who-director-general-s-opening- remarks-at-the-media-briefing-on-covid-19—11-march-2020. (accessed March 22, 2020).

[3] Coronavirus Update (Live): 629,450 Cases and 28,963 deaths from COVID-19 virus outbreak - worldometer n.d. https://www.worldometers.info/coronavirus. (accessed March 28, 2020).

[4] Zhou F, Yu T, Du R (2020). Clinical course and risk factors for mortality of adult inpatients with COVID-19 in Wuhan, China: a retrospective cohort study. The Lancet.

[5] Rubin GD, Ryerson CJ, Haramati LB (2020). The role of chest imaging in patient management during the COVID-19 pandemic: a multinational consensus statement from the Fleischner society. Radiology.

[6] Corman VM, Landt O, Kaiser M (2020). Detection of 2019 novel coronavirus (2019-nCoV) by real-time RT-PCR. Euro Surveillance.

[7] Fang Y, Zhang H, Xie J (2020). Sensitivity of chest CT for COVID-19: comparison to RT-PCR. Radiology.

[8] Ai T, Yang Z, Hou H (2020). Correlation of chest CT and RT-PCR testing in coronavirus disease 2019 (COVID-19) in China: a report of 1014 cases. Radiology.

[9] Zu ZY, Jiang MD, Xu PP (2020). Coronavirus disease 2019 (COVID-19): a perspective from China. Radiology.

[10] Wang Y, Dong C, Hu Y (2020). Temporal changes of CT findings in 90 patients with COVID-19 pneumonia: a longitudinal study. Radiology.

[11] Li X, Zeng W, Chen H (2020). CT imaging changes of corona virus disease 2019 (COVID-19): a multi-center study in Southwest China. J Transl Med.

[12] Chung M, Bernheim A, Mei X (2020). CT imaging features of 2019 novel coronavirus (2019-nCoV). Radiology.

[13] Bai HX, Hsieh B, Xiong Z (2020). Performance of radiologists in differentiating COVID-19 from viral pneumonia on chest CT. Radiology.

[14] Luo L, Luo Z, Jia Y (2020). CT differential diagnosis of COVID-19 and non-COVID-19 in symptomatic suspects: a practical scoring method. BMC Pulmonary Medicine.

[15] Tanaka N, Matsumoto T, Kuramitsu T (1996). High-resolution CT findings in community-acquired pneumonia. J Comput Assist Tomogr.

[16] Elicker BM, Webb WR (2013). Fundamentals of high-resolution lung Ct. Lippincott Williams & Wilkins. 1st edition ISBN:1451184085. Publisher: *Lippincott Williams & Wilkins*, (December 29, 2012) Reviewed in the United States*.*

[17] Grudzinska FS, Brodlie M, Scholefield BR (2020). Neutrophils in community-acquired pneumonia: parallels in dysfunction at the extremes of age. Thorax.

[18] Komiya K, Ishii H, Murakami J (2013). Comparison of chest computed tomography features in the acute phase of cardiogenic pulmonary edema and acute respiratory distress syndrome on arrival at the emergency department. Journal of thoracic imaging..

[19] Zompatori M, Ciccarese F, Fasano L (2014). Overview of current lung imaging in acute respiratory distress syndrome. Eur Respir Rev.

[20] Ferguson ND, Fan E, Camporota L (2012). The Berlin definition of ARDS: an expanded rationale, justification, and supplementary material. Intensive Care Med.

[21] Oikonomou A, Prassopoulos P (2011). CT imaging of blunt chest trauma. Insights into imaging.

[22] Wallis A, Spinks K (2015) The diagnosis and management of interstitial lung diseases. BMJ 350. https://www.google.com/url?sa=t&rct=j&q=&esrc=s&source=web&cd=&cad=rja&uact=8&ved=2ahUKEwii5JrZ-sDtAhUKSxUIHVqYDaUQFjAAegQIARAC&url=https%3A%2F%2Fwww.bmj.com%2Fcontent%2F350%2Fbmj.h2072%2Farticle-info&usg=AOvVaw2AvUa99WfYHLgyZ4jz9f0G. 10.1136/bmj.h2072 25952322

[23] Webb WR, Műller NL, Naidich DP (2008) High-resolution CT of the lung, 4th edn, ISBN:0781769094. Lippincott Williams & Wilkins. https://www.google.com/url?sa=t&rct=j&q=&esrc=s&source=web&cd=&cad=rja&uact=8&ved=2ahUKEwjt--LH-8DtAhXkoXEKHc2VDSIQFjAAegQIAhAC&url=https%3A%2F%2Fwww.amazon.com%2FHigh-Resolution-CT-Lung-Richard-Webb%2Fdp%2F0781769094&usg=AOvVaw1Oa1gCOAY8f7ArlHeyQbnQ.

[24] Marten K, Schnyder P, Schirg E (2005). Pattern-based differential diagnosis in pulmonary vasculitis using volumetric CT. AJR Am J Roentgenol.

[25] Lacasse Y, Girard M, Cormier Y (2012). Recent advances in hypersensitivity pneumonitis. Chest.

[26] Hirschmann JV, Pipavath SN, Godwin JD (2009). Hypersensitivity pneumonitis: a historical, clinical, and radiologic review. Radiographics.

[27] Mavridou D, Laws D (2004). Respiratory bronchiolitis associated interstitial lung disease (RB-ILD): a case of an acute presentation. Thorax.

[28] Holbert JM, Costello P, Li W (2001). CT features of pulmonary alveolar proteinosis. AJR Am J Roentgenol.

[29] Wittram C, Mark EJ, Mcloud TC (2003). CT-histologic correlation of the ATS/ERS 2002 classification of idiopathic interstitial pneumonias. Radiographics..

[30] Wolkove N, Marc Baltzan M (2009). Amiodarone pulmonary toxicity. Can Respir J.

[31] Jeong YJ, Kim KI, Seo IJ (2007). Eosinophilic lung diseases: a clinical, radiologic, and pathologic overview. Radiographics.

[32] Ohkoh T, Müller NL, Akira M (2000). Eosinophilic lung diseases: diagnostic accuracy of thin-section CT in 111 patients. Radiology.

